# Efficacy of modified technique for 1 cm core needle biopsy using a 2 cm cutting length with coaxial introducer needle

**DOI:** 10.1007/s00261-025-04904-0

**Published:** 2025-03-29

**Authors:** Hiroki Satomura, Yasushi Kimura, Masahisa Nakamura, Kosuke Tomotake, Koki Yamamoto, Kaishu Tanaka, Yusuke Ono, Hiroki Higashihara, Noriyuki Tomiyama

**Affiliations:** 1 Department of Diagnostic and Interventional Radiology, Osaka University Graduate School of Medicine, Osaka, Japan; 2 Department of High Precision Image-guided Percutaneous Intervention, Osaka University Graduate School of Medicine, Osaka, Japan; 3 Department of Diagnostic Radiology, Sakai City Medical Center, Osaka, Japan

**Keywords:** Coaxial introducer needle, Core needle biopsy, Image-guided biopsy, Percutaneous biopsy

## Abstract

**Purpose:**

This study evaluated the efficacy of a modified core needle biopsy using a 2 cm stroke length to improve tissue yield for molecular analyses in cancer genomics medicine.

**Methods:**

In vitro biopsy weight evaluations were performed using agarose gels of varying hardness. Ex vivo biopsy weight evaluations were conducted using chicken liver, gizzard, breast, and pork loin tissues. Furthermore, in vivo experiments were performed using murine subcutaneous tumor models. The modified method involved using a coaxial introducer needle to maintain a 1 cm notch length while employing a 2 cm stroke length to increase tissue yield. Biopsy performance parameters, including sample weight and DNA yield, were compared between the modified and conventional methods. The firing speed of the biopsy needles with 1 cm and 2 cm stroke lengths was also measured using a high-speed camera.

**Results:**

The modified technique significantly increased the sample volume across all agarose gel concentrations (9.5–13% increase, P < 0.05). Ex vivo tests revealed significantly higher tissue yields in most samples, except for chicken gizzards. In vivo, the modified method produced significantly larger tissue samples (4.6 mg vs. 2.9 mg, P < 0.001) and higher DNA yield (8.2 ng vs. 6.9 ng, P < 0.05). In addition, the biopsy needle’s firing speed was 1.4–1.8 times faster with the 2 cm stroke length than 1 cm stroke.

**Conclusion:**

The modified biopsy technique using a 2 cm stroke length significantly improved tissue yield, enhancing the quality of biopsy samples for molecular analyses in precision medicine.

## Introduction

Percutaneous image-guided biopsy is well established in clinical practice owing to its accuracy, low complication rate, and minimally invasive approach [1–3]. Furthermore, with recent advances in cancer genomics medicine, percutaneous biopsy has become essential [3]. The role of biopsy has evolved from just diagnosing and classifying malignancies by histology. Genetic analyses of biopsy samples enable the identification of specific molecular and genetic alterations in tumors, facilitating patient stratification based on tumor profiles and supporting personalized treatment with targeted therapies [3–5]. Moreover, biopsies are also conducted at multiple time points to evaluate the response to treatment, assess biomarker status, and determine post-treatment management [6]. Hence, there is an increasing amount of tissue required for molecular pathological analysis in precision medicine.

Various types of biopsy devices are available, including notch-type (semi-automatic and automatic), end-fire, and non-notch (full-core) devices, each with distinct mechanisms for tissue acquisition. Sample length varies depending on the device and procedural settings, but the most commonly used lengths are approximately 1 cm or 2 cm. Among these, semi-automatic biopsy needles are commonly used for percutaneous biopsy. These needles allow the selection of a sample length of approximately 1 cm or 2 cm at the lesion extraction site by adjusting the needle stroke length according to the lesion size. A 1 cm stroke length is often chosen for small lesions; however, shorter sample lengths may sometimes result in inadequate tissue yield [7]. Additionally, biopsy of smaller lesions tends to have higher sampling error rates and longer procedure times due to the challenges of accurately targeting the lesion and the difficulty in obtaining a sufficient amount of tissue per attempt [8, 9]. Therefore, strategies to maximize the sample yield per biopsy are needed.

Several methods can be used to increase specimen volume. First, using a larger needle or increasing the number of biopsies can be effective [10–12]; nonetheless, these approaches may increase the risk of complications [13]. One approach is to use improved needles, such as steerable [14] or aspiration-type biopsy needles [15, 16]. Additionally, there have been reports on the use of navigation systems [17] and robot-assisted biopsy [18, 19]. Another approach involves the use of non-notch (full-core) biopsy devices, which are designed to obtain intact cylindrical tissue cores, potentially improving sample volume and integrity. Although these devices have shown promise, they are not yet widely adopted for percutaneous image-guided biopsies, and further studies are needed to evaluate their clinical utility. To date, no standardized method has been established for increasing specimen volume in percutaneous biopsy.

Given that the trigger mechanism of the semiautomatic biopsy needle is spring-loaded, it is thought that the longer the stroke length, the stronger the firing of the cannula. Thus, we hypothesized that using a stroke length of 2 cm would yield more tissue. This approach involves adjusting the actual lesion extraction site, specifically the notch part protruding from the tip of the coaxial introducer needle, to match the lesion size, making it applicable even for small lesions. In this study, we aimed to evaluate the efficacy of a modified technique for a 1 cm core needle biopsy using a 2 cm cutting length with a coaxial introducer needle.

## Materials and methods

Ethical approval was not required from our institution for this in vitro or ex vivo study. In vivo experiments using mice were conducted according to a protocol reviewed and approved by the Institutional Animal Care and Use Committee of our institution (04-107-004).

### Modified technique for 1 cm core needle biopsy

**Fig. 1:**
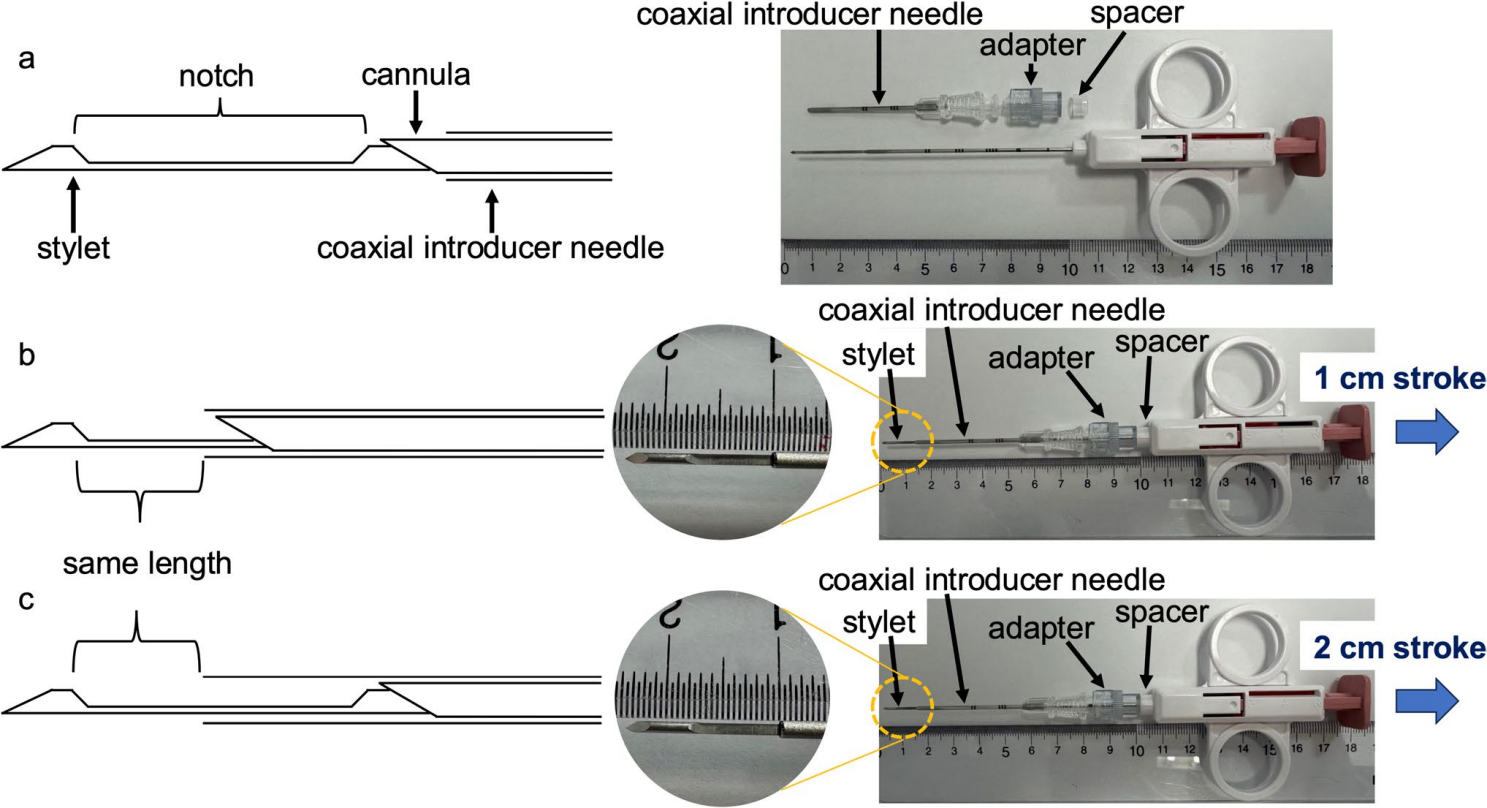
Schematic and photographic representation of the biopsy system used in this study. (**a**) Illustration of the semi-automatic biopsy needle (18G, SuperCore™) commonly used in percutaneous image-guided procedures. (**b**) Conventional biopsy method using a 1 cm stroke length, shown with both schematic illustration and actual photograph. (**c**) Modified biopsy method using a 2 cm stroke length with a coaxial introducer needle, shown with both schematic illustration and actual photograph. The modified method includes the use of an adapter and spacer to maintain a consistent notch length protruding from the coaxial introducer needle

In this study, a coaxial system comprising the SuperCore™ semi-automatic biopsy needle (18G, Argon Medical Devices, TX, USA) and an introducer needle was used for all experiments. We selected this biopsy needle because it is the most commonly available at our institution, ensuring feasibility and consistency across experiments. We adjusted the notch length protruding from the tip of the coaxial introducer needle to be consistent (1 cm) by using an adapter and a spacer. The spacer was created by cutting a plastic tube originally provided as a protective cover for the biopsy needle, ensuring that the notch length was identical and stable for both the conventional and modified methods. This setup prevented any unintended shift in the relative position of the introducer and biopsy needles during insertion and firing, maintaining a consistent protrusion length (Fig. 1). The stroke length was set to 2 cm or 1 cm, with the biopsy using a 2 cm stroke length designated as the modified method, and the biopsy using a 1 cm stroke length designated as the conventional method.

### In vitro experiments

An agarose gel, whose hardness could be adjusted by changing its concentration, was used. Based on reports that the hardness of the liver and brain corresponds to 1% agarose gel, and that of the heart and kidneys to 2% agarose gel, agarose gels with concentrations ranging from 0.5 to 3% were employed [20]. Agarose LE (FUJIFILM Wako Pure Chemical Corporation, Osaka, Japan) was added to 50 mL of Dulbecco's Modified Eagle Medium (DMEM) (FUJIFILM Wako Pure Chemical Corporation, Osaka, Japan) at concentrations of 0.5, 1, 1.5, 2, 2.5, and 3% and mixed thoroughly. The hardness of the agarose gel at each concentration was measured using TexoGraph (JAPAN FOOD R&D INSTITUTE, Osaka, Japan). A load-deformation curve was generated, and the initial slope was analyzed to compare the rigidity. The prepared 0.5–3% agarose gels were subjected to a total of 120 biopsies, with 60 performed using the modified method and 60 using the conventional method. The weight of the tissue obtained per biopsy was measured using an electronic balance (IUW-200D; AS ONE CORPORATION, Osaka, Japan).

### Ex vivo experiments

Chicken liver, gizzard, breast, and pork loin were utilized as ex vivo models in this study. These tissues were sourced fresh from a local grocery store and used immediately for experimentation. As the organs were derived from commercially available food products rather than live animals, no additional ethical approval was necessary. These tissues were manually stabilized by gently holding them to prevent movement during biopsy, ensuring that excessive pressure was avoided to prevent tissue compression. Each tissue type was subjected to a total of 40 biopsies, with 20 performed using the modified method and 20 using the conventional method. The weight of the tissue obtained per biopsy was measured using an electronic balance.

### In vivo experiments

The murine colorectal carcinoma cell line (CT26) was used in this study. The cells were cultured in Dulbecco’s modified Eagle medium (DMEM, Fujifilm-Wako Pure Chemicals, Osaka, Japan), supplemented with 10% fetal bovine serum (FBS, Nichirei Bioscience Inc., Tokyo, Japan) and antibiotics (Antibiotic–Antimycotic; Cytiva, Tokyo, Japan), at 37 °C and 5% CO_2_. Eight-week-old female BALB/cCrSIc mice (Japan SLC, Inc., Shizuoka, Japan) were subcutaneously injected with CT26 cells (1 × 10^6^ cells/0.1 mL) on both sides of the lower back. The mice were then kept for a few weeks to allow the tumors to grow to a minimum length of approximately 8 mm. Overall, 18 tumors from nine mice were analyzed. Biopsies were performed without image guidance; the subcutaneous tumors were visually identified and directly punctured. To ensure consistency, the tumors were gently stabilized by hand to prevent movement during the procedure, while avoiding excessive pressure that could potentially affect the sample yield. For each of the 18 tumors, both the modified and conventional methods were alternately used three times each, ensuring direct comparison within the same tumor group. The total weights of the samples collected from the three biopsies were measured using an electronic balance. DNA was extracted and purified from each sample using the standard protocol of the ISOSPIN Tissue DNA kit (NIPPON GENE Co., Ltd., Tokyo, Japan). The extracted DNA was quantified using Thermo Scientific™ NanoDrop™ One C (Thermo Fisher Scientific K.K., Tokyo, Japan), and the average value was calculated after three measurements for each sample.

### Measurement of the firing speed of the biopsy needle

The firing of the biopsy needle was recorded using a high-speed camera (VW-9000TM; KEYENCE, Osaka, Japan). The shutter speed was set to 1/16,000 s, and the frame rate was 5996.4 frames per second (fps). Recordings were made for the 1 cm and 2 cm stroke lengths, and the actual firing speeds of the cannulas were compared. Three semi-automatic biopsy needles were used in this experiment: SuperCore ™ (Argon Medical Devices, TX, USA), Mission ™ (BARD, AZ, USA), and AprioCore^®^ plus (AprioMed, Uppsala, Sweden).

### Statistical analysis

For each experiment, the mean and standard deviation (SD) of the sample weight and DNA quantity were calculated. The differences in sample weight and DNA quantity and their 95% confidence intervals (CIs) were compared using the Student's t-test. Comparisons of the weights in the in vitro and ex vivo experiments were conducted using an unpaired one-sided t-test. A paired one-sided t-test was performed for the in vivo experiments. Statistical analyses were conducted using R software (version 4.2.0), and statistical significance was set at P < 0.05.

We considered measuring the actual length of the core samples; however, transferring the specimens from the biopsy needle notch led to deformation, making precise length measurement difficult. Therefore, we used sample weight as a more reliable parameter for comparing specimen volume. In in vivo experiments, DNA yield was also assessed, as it is clinically more relevant than sample weight and enhances the accuracy of comparisons.

## Results

### In vitro experiments

**Fig. 2:**
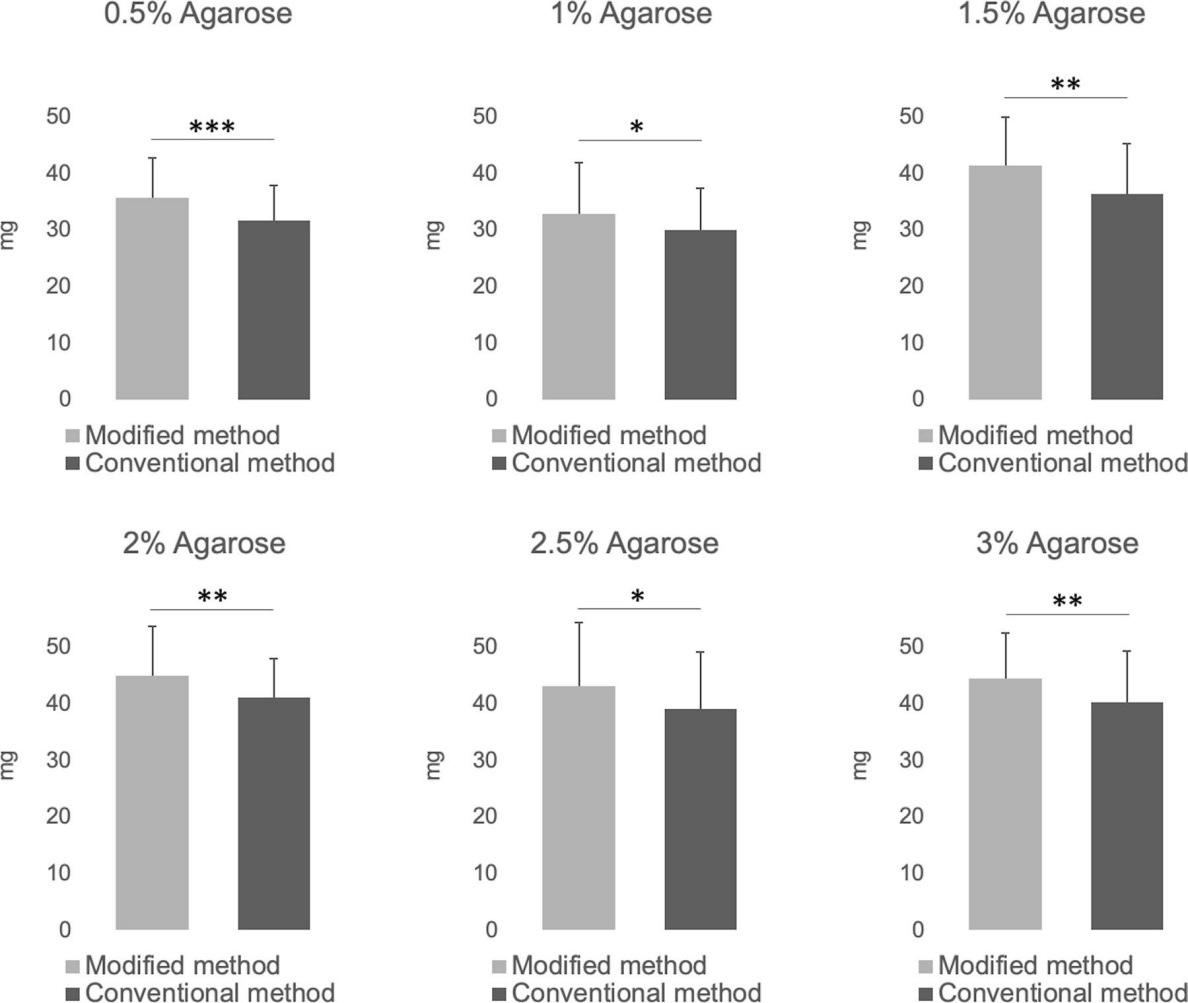
Comparison of sample volumes obtained from agarose gels with different concentrations using the modified and conventional biopsy methods. Bar graph showing the mean sample volumes collected from agarose gels of varying concentrations (0.5% to 3.0%) using the modified 2 cm stroke length method and the conventional 1 cm stroke length method. The modified method consistently yielded larger sample volumes across all concentrations compared to the conventional method. *P < 0.05, **P < 0.01, ***P < 0.001, *NS* non-significant

In all agarose gel concentrations, the modified 1 cm needle biopsy yielded a significantly larger sample volume (Fig. 2). A comparison of the mean values revealed increased sample volume, ranging from a minimum of 9.5% to a maximum of 13%. The rigidity of the agarose gels increased with concentration, with values of 29 ± 2.4 gf/mm for 0.5%, 115 ± 2.7 gf/mm for 1%, 210 ± 14 gf/mm for 1.5%, 349 ± 29 gf/mm for 2%, 498 ± 25 gf/mm for 2.5%, and 638 ± 32 gf/mm for 3%.

### Ex vivo experiments

**Fig. 3:**
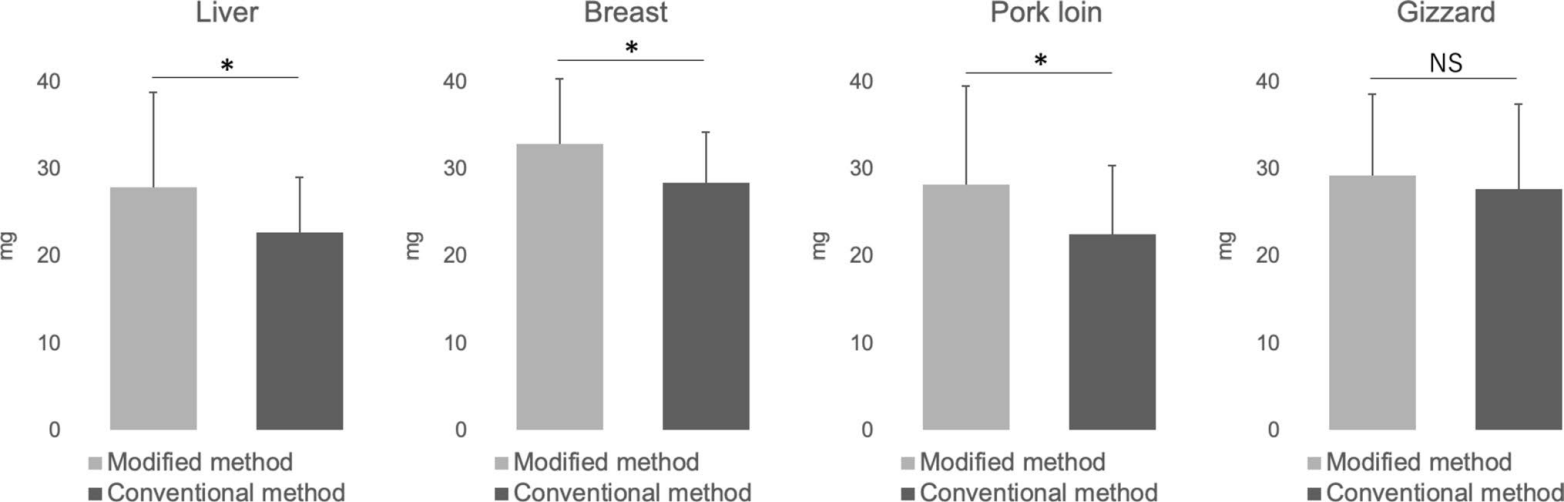
Comparison of sample volumes obtained from ex vivo organs using the modified and conventional biopsy methods. Bar graph showing the mean sample volumes collected from chicken liver, chicken breast, pork loin, and gizzard using the modified 2 cm stroke length method and the conventional 1 cm stroke length method. The modified method yielded significantly larger samples in most tissues, except for the gizzard, where the difference was not statistically significant. *P < 0.05, **P < 0.01, ***P < 0.001, *NS* non-significant

The ex vivo results are shown in Fig. 3. For chicken liver, chicken breast, and pork loin, the sample amount was significantly increased using the modified 1 cm method (23% increase; P < 0.05, 16% increase; P < 0.05, 25% increase; P < 0.05, respectively). While there was a tendency for the sample amount to be higher with the modified 1 cm method in chicken gizzards, the difference was not statistically significant (P = 0.31).

### In vivo experiments

**Fig. 4:**
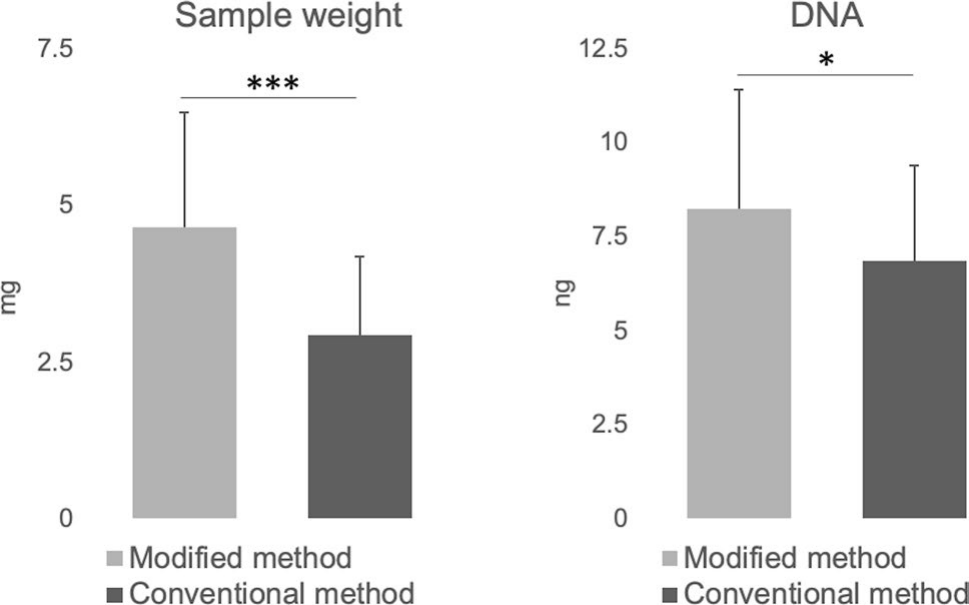
Comparison of sample weight and DNA yield from subcutaneous mouse tumors using the modified and conventional biopsy methods. Bar graph displaying the mean sample weight (mg) and DNA yield (ng) collected from subcutaneous tumors in mice. Biopsies were performed using the modified 2 cm stroke length method and the conventional 1 cm stroke length method. The modified method resulted in significantly larger sample weights and higher DNA yields compared to the conventional method. *P < 0.05, **P < 0.01, ***P < 0.001, *NS* non-significant

The tumors had an average major axis of 13.4 mm (range 8–18 mm) and a minor axis of 10.8 mm (range 5–15 mm). The sample weight was significantly higher with the modified method, yielding 4.6 ± 1.8 mg compared with 2.9 ± 1.2 mg with the conventional method (P < 0.001). Similarly, the DNA yield was significantly increased using the modified method, measuring 8.2 ± 3.2 ng compared with 6.9 ± 2.5 ng with the conventional method (P < 0.05) (Fig. 4).

### Measurement of the firing speed of the biopsy needle

**Fig. 5:**
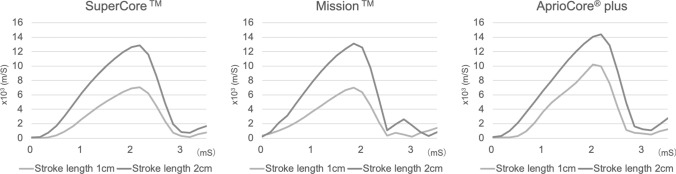
High-speed imaging of biopsy needle firing speeds with 1 cm and 2 cm stroke lengths. High-speed camera capture comparing the firing speeds of three different semi-automatic biopsy needles: SuperCore™, Mission™, and AprioCore^®^ plus. The firing velocity of each needle's cannula was recorded at stroke lengths of 1 cm and 2 cm using a high-speed camera. The results demonstrated that the cannula firing speed was 1.4–1.8 times faster with the 2 cm stroke length compared to the 1 cm stroke length across all needle types

The firing speed results are shown in Fig. 5. For all three biopsy needles, the firing speed of the cannula was faster with a 2 cm stroke length. Notably, compared with the 1 cm stroke length, the 2 cm stroke length resulted in a firing velocity that was 1.4–1.8 times faster.

## Discussion

The results of this study demonstrated that modifying the semiautomatic biopsy technique by utilizing a 2 cm stroke length while maintaining the notch length at 1 cm with a coaxial introducer needle significantly increased the sample volume, both in vitro, ex vivo and in vivo. This finding has several implications for clinical practice, particularly regarding precision medicine, which has an increasing demand for larger, higher-quality biopsy specimens for molecular and genetic analyses.

In vitro experiments using agarose gels of varying concentrations revealed that the modified biopsy method consistently yielded larger sample volumes across all gel hardness levels. This suggests that the modified technique is applicable to tissues with different consistencies, making it potentially useful for the biopsy of various tumors. Ex vivo experiments further validated these findings, showing that the modified method significantly increased the sample yield in most tissue types, except for chicken gizzards, where the difference was not statistically significant. This variation could be attributed to the toughness of gizzard tissue, which may require further refinement of the technique or an alternative needle design. Nevertheless, these results suggest that the modified technique can be applied to different tissue types with similar increases in sample yield.

In vivo experiments with murine tumor models demonstrated that the modified method increased sample weight and DNA yield, which is essential for molecular analyses requiring high-quality DNA. Genetic testing platforms have varying DNA requirements; for example, Next-Generation Sequencing (NGS) hotspot panel tests may require as little as 10 ng of DNA [21–23]. While the obtained DNA yield of 8.2 ng may seem lower than the requirements for human tumor samples, it should be interpreted considering key biological differences between murine subcutaneous tumors and human tumors, such as cellular density, tumor structure, and necrosis. The significant increase in DNA yield achieved with the modified method compared to the conventional approach highlights its potential to improve biopsy sample quality and quantity. This suggests that the modified technique could be beneficial for precision medicine applications, particularly in longitudinal studies requiring repeated biopsies to monitor disease progression or treatment response. Although this study did not assess safety parameters, the procedural aspects were consistent with standard biopsy practices, indicating that it can be implemented clinically without increasing invasiveness.

The increased sample volume with the 2 cm stroke length is likely due to the faster firing speed of the biopsy cannula. A higher firing speed improves tissue cutting and extraction while reducing tissue deformation and fragmentation, thereby improving sample quality [24]. Previous study suggests that a firing speed of at least 8 m/s is considered optimal for achieving sufficient tissue yield and improving procedural success [25]. Additionally, higher firing speeds have been shown to enhance tissue acquisition without compromising integrity [24]. These findings support the potential of the 2 cm stroke length, which achieved firing speeds of at least 8 m/s across all biopsy needles, to provide higher-quality biopsy samples. Still, while increased speed enhances sample yield, it is crucial to monitor whether this might increase the risk of tissue damage, especially in more fragile tissues. The clinical significance of increased firing speed and its potential impact on patient safety and procedural outcomes requires further investigation.

In addition, it is important to note that the difference in firing speed between the 1 cm and 2 cm stroke lengths varied depending on the type of biopsy needle used. In particular, the AprioCore^®^ plus needle with a 1 cm stroke length exhibited a slightly higher firing speed compared to other needles with the same stroke length. This suggests that needle-specific characteristics, such as internal friction, spring tension, and structural design, may influence firing performance. These factors may also affect biopsy outcomes, highlighting the need for further studies to evaluate various needle designs. However, it remains unclear how the variable fire rate of the AprioCore^®^ plus needle would influence the performance of the modified technique. Since this study did not directly evaluate this device, future research is needed to determine whether the modified technique provides similar benefits when applied to biopsy needles with variable firing speeds.

This study has some limitations. First, it primarily focused on in vitro, ex vivo and animal models. These results are promising; however, clinical trials with human participants are required to confirm the efficacy and safety of the modified biopsy technique in clinical settings. Additionally, only a single type of biopsy needle was tested in this study, and further research is needed to determine whether similar results can be achieved with other needle types. Moreover, it remains unclear whether the slight increase in DNA yield achieved with the modified method contributes to the improved success rate of genetic analyses in clinical settings. Furthermore, this study did not assess the potential impact of the modified method on complication rates, which are critical factors in percutaneous biopsy procedures. Future studies should evaluate whether the increased sample volume achieved with the modified technique results in a higher risk of complications, such as bleeding, infection, or tissue damage.

Additionally, the benefit of the modified technique is expected to be most relevant for sampling small targets, particularly those ≤ 1 cm in size. For lesions > 2 cm in diameter, proceduralists may prefer using the full 2 cm throw without an adapter or spacer to maximize tissue acquisition. Therefore, the applicability of this technique may be limited in larger lesions where a conventional 2 cm stroke length can be fully utilized.

In conclusion, the modified 1 cm core needle biopsy with a 2 cm stroke length offers a promising approach to increasing the tissue yield in percutaneous biopsy. This technique may be critical in improving the quality and quantity of biopsy samples used for molecular analyses, particularly in the era of precision medicine.

## Data Availability

The authors declare that the data supporting the findings of this study are available within the paper. Should any raw data files be needed in another format they are available from the corresponding author upon reasonable request.
